
JOURNAL INFORMATION
==============================
NLM Title Abbreviation: Behav Neurol
Journal ID: Behav Neurol
NLM Journal ID: 8914585
Journal ID: BN

Behavioural Neurology

ISSN: 0953-4180
EISSN: 1875-8584
Publisher: Wiley

ARTICLE INFORMATION
==============================
PMCID: PMC5215437
PMID: 22713429
DOI: 10.3233/BEN-2012-129013
Article ID: 584176
Article version: 1
Subjects: Other

Towards a Left MTL Specialization for Arbitrary-Associative Learning? A Multiple-Case Study

Jonin Pierre-Yves 1 *
Noel Audrey 2
Trebon Pascale 1
Vichard Hélène 1
Merck Catherine 1
Belliard Serge 1 3
1 Department of Neurology Pontchaillou University Hospital Rennes France
2 Centre de Recherches Psychologie Cognition and Communication (EA 1285) Rennes 2 University Rennes France
3 Inserm Unit U1077 Neuropsychologie et neuroanatomie fonctionnelle de la mémoire humaine Caen France
*Pierre-Yves Jonin: pierreyves.jonin@chu-rennes.fr
Print publication date: 2013
Volume: 26
Issue: 3
First page: 211
Last page: 213
Received 2012 May 21; Accepted 2012 May 21
Copyright: Copyright © 2013 Hindawi Publishing Corporation and the authors.
Copyright year: 2013
License: This is an open access article distributed under the Creative Commons Attribution License, which permits unrestricted use, distribution, and reproduction in any medium, provided the original work is properly cited.
License URL: https://creativecommons.org/licenses/by/3.0/

Keywords: Medial temporal lobe, associative learning, lateralization

==============================
Behavioural Neurology 26 (2013) 211–213
DOI 10.3233/BEN-2012-129013
IOS Press

### Towards a left MTL specialization for arbitrary-associative learning? A multiple-case study

Pierre-Yves Jonin[a,*], Audrey Noel[b], Pascale Trebon[a], Hélène Vichard[a], Catherine Merck[a] and Serge Belliard[a,c]
[a]*Department of Neurology, Pontchaillou University Hospital, Rennes, France*
[b]*Centre de Recherches Psychologie, Cognition and Communication (EA 1285), Rennes 2 University, Rennes, France*
[c]*Inserm Unit U1077, Neuropsychologie et neuroanatomie fonctionnelle de la mémoire humaine, Caen, France*

Keywords: Medial temporal lobe, associative learning, lateralization

### 1. Introduction

Since the famous case of HM provided unquestionable evidence for the involvement of medial temporal lobe (MTL) structures in memory, a dominant neuropsychological model raised up in the 1970's. Brenda Milner proposed that "*the most significant variable* (*to explain post-surgery memory outcome in temporal lobe epilepsy*) *is the verbal or non-verbal character of the material to be retained*" [1]. This model predicts that verbal and non-verbal memories are homogenous and independent systems, respectively relying upon left and right temporal lobes.

Yet, series of cases of amnesia following unilateral temporal lobectomy have been documented, drawing a more complex picture [2]. Moreover, cases of poorer verbal memory outcome after right TL surgery had been reported [3], and other studies underlined deficits in spatial learning after left surgery. Taken together, those data strongly challenges the dominant material-specific view.

In line with those challenging data, other authors suggested that beyond material-specificity, task-specificity may be a core variable. Indeed, Saling et al. [4] demonstrated that only acquisition of unrelated word pairs accurately predicted left MTL dysfunction, whereas prose recall or acquisition of related word pairs failed to differentiate between right and left patients. However, associative learning tasks most often use word pairs, whereas other material like object-location associations did not consistently discriminate right and left MTL dysfunction [5].

The question then arises as to whether the lateralization of MTL dysfunction could be accurately predicted following material-specific model, or task-specific model. Moreover, given the frequent use of words as support for associative learning, any specificity of left MTL for associative memory could reflect a material bias. We therefore aimed at verify whether arbitrary-associative learning could discriminate between right and left temporal lobectomy, using both verbal and non-verbal materials, within the framework of a single learning task.

### 2. Materials and methods

### 2.1. Participants

We identified retrospectively temporal lobe epilepsy (TLE) patients who underwent an unilateral standard en

*Corresponding author: Pierre-Yves Jonin, Department of Neurology, Pontchaillou University Hospital, Rennes, France. E-mail: pierreyves.jonin@chu-rennes.fr.

ISSN 0953-4180/13/$27.50 © 2013 – IOS Press and the authors. All rights reserved

Table 1
Experimental memory procedure: MTL resection participants and control group performances

| | Immediate Trial 1 | | | Immediate Trial 2 | | | Immediate Trial 3 | | | Immediate Trial 4 | | | Delayed Trial | | |
|---|---|---|---|---|---|---|---|---|---|---|---|---|---|---|---|
| | Words | Loc. | *Asso* | Words | Loc. | *Asso* | Words | Loc. | *Asso* | Words | Loc. | *Asso* | Words | Loc. | *Asso* |
| **Right MTL** | | | | | | | | | | | | | | | |
| Case 1 | 7 | 5 | 2 | 6 | 6 | 4 | 6$^{(a)}$ | 7 | 6 | 4$^{(b)}$ | 8 | 8 | 4$^{(a)}$ | 8 | 5 |
| Case 2 | 7 | 1$^{(a)}$ | 0 | 7 | 5 | 4 | 7 | 5$^{(a)}$ | 4 | 8$^{(a)}$ | 6$^{(a)}$ | 7 | 8 | 7 | 7 |
| Case 3 | 5 | 4 | 2 | 7 | 6 | 7 | 7 | 7 | 7 | 8 | 8 | 7 | 7 | 6 | 6 |
| **Left MTL** | | | | | | | | | | | | | | | |
| Case 1 | 4 | 2 | 0$^{(a)}$ | 3$^{(a)}$ | 2 | 1 | 5$^{(a)}$ | 7 | 0 | 6$^{(b)}$ | 7 | 1$^{(b)}$ | 5$^{(b)}$ | 4$^{(a)}$ | 2$^{(a)}$ |
| Case 2 | 5 | 4 | 1 | 5 | 5 | 1 | 6$^{(a)}$ | 5 | 3 | 6$^{(b)}$ | 6 | 1$^{(a)}$ | 4$^{(a)}$ | 6 | 1$^{(a)}$ |
| Case 3 | 5 | 3 | 0 | 4$^{(a)}$ | 3 | 0 | 5$^{(a)}$ | 2 | 0 | 5$^{(a)}$ | 5 | 1 | 0$^{(a)}$ | 3 | 0$^{(a)}$ |
| **Controls** | | | | | | | | | | | | | | | |
| Mean | 6,0 | 5,1 | 4,0 | 4,0 | 7,8 | 6,0 | 7,8 | 7,1 | 6,5 | 8,0 | 7,6 | 7,6 | 7,6 | 7,4 | 6,9 |
| SD | 1,3 | 2,9 | 2,6 | 2,6 | 0,7 | 2,3 | 0,5 | 1,5 | 2,3 | 0,0 | 0,5 | 0,7 | 0,5 | 0,7 | 1,6 |
| Median | 6,0 | 6,0 | 3,0 | 3,0 | 8,0 | 6,5 | 8,0 | 8,0 | 8,0 | 8,0 | 8,0 | 8,0 | 8,0 | 7,5 | 7,5 |

(Words = word recall condition; Loc. = location recall condition; *Asso.* = associative recall condition; $^{(a)} = p < 0{,}05$; $^{(b)} = p < 0{,}01$).

bloc resection of MTL structures (including amygdala, hippocampus, temporal pole, entorhinal & perirhinal cortices). Six patients (3 right "RMTL" and 3 left MTL "LMTL" resection) were included on the basis of (1) an extensive pre- & post-surgery neuropsychological assessment with IQ > 80 and MQ > 75, (2) a delay from surgery superior to 18 months (range 19–92 months), (3) absence of seizures post surgery, (4) no extratemporal abnormalities on MRI. All patients had to be right handers and French-native speakers. A group of N = 19 healthy control participants were recruited, and paired-matched to each TLE participant.

### 2.2. *Material*

A 41-squares grid was presented, with 8 abstracts words located in 8 eight different squares. Participants were invited to encode the words and their locations. Words were matched together for lexical frequency, number of syllabs and a very poor degree of imageability.

### 2.3. *Procedure*

An encoding trial controlled for reading and location abilities. Thereafter, participants were presented the learning grid for a 45 seconds free encoding trial, followed by the immediate recall of the words, then the locations, and finally an immediate recall of the associations between words and locations. Four immediate recall procedures were administrated, along with a delayed recall trial. Importantly, given the expected worse performance of left TLE participants for words learning, associative recall was two-staged. First, participants were given visually the words they recalled in the preceding words recall trial, and they had to write down those items in the right locations. Secondly, remaining non recalled words were given and the same was asked to the participants. This allowed us to avoid any artefactual associative deficit actually due to a poor word-recall performance.

### 2.4. *Statistics*

Following Crawford and Howell [6] single case methodology, we adopted a conservative statistical approach limiting type I errors, by applying their modified one tailed t-tests procedure. This procedure further provides an estimated percentage of the normal population falling below one single case's score (i.e. 5% whenever a significant difference was reached at 0.05 chance level) as well as an effect size of the difference observed.

### 3. Results

All experimental data are presented in Table 1.

### 3.1. *Right MTL patients* (*RMLT*)

Two participants were impaired either for immediate or delayed recall of single items (i.e. words or locations, effect sizes ranging from −1,810 to −7,600). However, no deficit was observed during associative recall condition.

### 3.2. *Left MTL patients* (*LMTL*)

Single items recall conditions (words or locations) yielded significant impairments for the 3 patients during delayed trials, whereas immediate trials showed variable deficits, more pronounced as expected for words condition (effect sizes ranging from −2,000 to −20,000). By contrast, associative recall conditions yielded a significant deficit for all patients and for each trial with the exception of trial 1 for cases 2 & 3 (effect sizes ranging from −2,000 to −7,222). Moreover, no learning effect occurred in left participants, thus contrasting with right participants.

To sum up, a sharp contrast emerged between right and left MTL resection participants only for the associative recall conditions, where all RMTL remained within normal range, whereas LMTL demonstrated constant impairment and no evidence of learning effect.

### 4. Discussion

Our data highlight the superiority of an associative learning procedure in differentiating right from left MTL resection. Indeed, despite the verbal and non verbal character of the words and locations used in our study, learning those single items did not allow any accurate lateralization of lesion. By contrast, only LMTL failed to learn and recall associations between words and their locations.

At a cognitive level, those results confirm previous work suggesting that the processes required to encoding and storing new associations critically depend on left medial temporal lobe structures [7]. To our knowledge, this is the first study to provide such evidence with verbal-spatial associations. Previous reports [5,8,9] already suggested a left specialization for auditory-spatial and object-location associations, which strengthens our findings.

At a theoretical level, our data clearly speak for a shift from the material-specific model that goes on guiding many clinicians in the field. Along with Michael Saling and coauthors's view [10], our data speak for an alternative task-specific view. According to this model, arbitrary-associative learning and retention would critically depend on the interactions between left perirhinal cortex and hippocampus. By contrast, learning and retention of semantic associations or of single meaningful items would rely upon more lateral structures like the fusiform gyrus. As a consequence, only tasks involving arbitrary associative learning would accurately predict MTL dysfunction. Our results further suggest that this kind of learning could be more lateralized to the left hemisphere, which remains to be asserted with experimental paradigms involving associative recall and/or recognition of non-verbal items.

Given the importance of associative memory in daily life, future research needs to include various associative tasks to confirm their specificity for MTL functioning, then to prospectively assess their predictive power for cognitive outcome and quality of life.

### References

[1] B. Milner, Memory and the medial temporal regions of the brain, in: *Biology of Memory*, K.H. Pribram and D.E. Broadbent, eds, New York: Academic Press, 1970, pp. 29–50.

[2] N. Kapur and M. Prevett, Unexpected amnesia: are there lessons to be learned from cases of amnesia following unilateral temporal lobe surgery? *Brain* **126** (2003), 2573–2585.

[3] U. Gleissner, C. Helmstaedter, J. Schramm and C.E. Elger, Memory outcome after selective amygdalohippocampectomy: a study in 140 patients with temporal lobe epilepsy, *Epilepsia* **43** (2002), 87–95.

[4] M.M. Saling, M.F. O'Shea, D.L. Weintrob, A.G. Wood, D.C. Reutens and S.F. Berkovic, Medial and lateral contributions to verbal memory: evidence from temporal lobe epilepsy, in: *Frontiers of Memory*, A. Yamadori, R. Kawashima, T. Fujii and K. Suzuki, eds, Sendai: Tohoku University Press, 2002, pp. 151–158.

[5] A. Incisa della Rocchetta, S. Samson, N. Ehrle, M. Denos, D. Hasboun and M. Baulac, Memory for visuospatial location following selective hippocampal sclerosis: the use of different coordinate systems, *Neuropsychology* **18**(1) (2004), 15–28.

[6] J.R. Crawford and D.C. Howell, Comparing an individual's test score against norms derived from small samples, *The Clinical Neuropsychologist* **12** (1998), 482–486.

[7] D.L. Weintrob, M.M. Saling, S.F. Berkovic and D.C. Reutens, Impaired verbal associative learning after resection of left perirhinal cortex, *Brain* **130** (2007), 1423–1431.

[8] C. Lancelot, P. Ahad, M. Noulhiane, D. Hasboun, M. Baulac and S. Samson, Loss of memory for auditory-spatial associations following unilateral medial temporal-lobe damage, *Neuropsychologia* **43**(13) (2005), 1975–1982.

[9] R.C. Kessels, L.J. Kappelle, E.H.F. de Haan and A. Postma, Lateralization of spatial-memory processes: evidence on spatial span, maze learning, and memory for object locations, *Neuropsychologia* **40** (2002), 1465–1473.

[10] M.M. Saling, Verbal memory in mesial temporal lobe epilepsy: beyond material specificty, *Brain* **132** (2009), 570–582.
